# Rationality and Irrationality in Ryke Geerd Hamer's System for Holistic Treatment of Metastatic Cancer

**DOI:** 10.1100/tsw.2005.16

**Published:** 2005-01-28

**Authors:** Søren Ventegodt, Niels Jørgen Andersen, Joav Merrick

**Affiliations:** ^1^ The Quality of Life Research Center, Teglgårdstræde 4, DK-1452 Copenhagen K, Denmark; ^2^ The Research Clinic for Holistic Medicine, Teglgårdstræde 8, DK-1452 Copenhagen K, Denmark; ^3^ Norwegian School of Management, Sandvika, Norway; ^4^ The Scandinavian Foundation for Holistic Medicine, Sandvika, Norway; ^5^ National Institute of Child Health and Human Development, Division for Mental Retardation, Ministry of Social Affairs, Jerusalem and Zusman Child Development Center, Division of Pediatrics and Community Health, Ben Gurion University, Beer-Sheva, Israel

**Keywords:** quality of life, QOL, human development, holistic medicine, public health, holistic health, cancer, Ryke Geerd Hamer, alternative medicine, complementary medicine, consciousness-based medicine, Denmark

## Abstract

The aim of this paper is to examine if the “medical laws” found by the German physician Ryke Geerd Hamer are substantiated by contemporary holistic medical theory. He developed a psychosomatic theory after a personal emotional trauma that he believed resulted in his subsequent development of a testicular cancer. From our analysis, it is clear that the two most fundamental principles of Hamer's work, the psychosomatic “iron law of cancer” (Hamer's first “law”) and the principle of pathogenesis being reversed into salutogenesis (Hamer's second “law”), are well-established principles of holistic medicine today. Hamer's understanding of symbols in medicine, virus and bacteria, and the evolutionary process itself (Hamer's third, fourth, and fifth “law”) differs a great deal from both traditional and contemporary holistic medical theory and we did not find them substantiated. Hamer's understanding of cancer metastasis was built on these failing principles and therefore not substantiated either. Altogether, it seems that Hamer's thinking was basically sound as the most fundamental principles of his work were built on an understanding very similar to holistic medical thinkers of today. We found his postulate that metastatic cancer patients can be healed or their health improved by using his system of holistic medicine likely to be true, at least for some motivated patients. This must be tested scientifically, however, before being accepted. His presentation of his system and work has been idiosyncratic and highly provocative, which has alienated him from the whole medical community.

## INTRODUCTION

The aim of this paper was to review, from a theoretical perspective, the Hamer system of holistic cancer medicine[1,2,3,4] that he called “the new medicine”, with the purpose of guiding both the physician and the patient in the very difficult area of holistic treatment of cancer. We all want the best for our patients and we believe that the solution is a wise combination of conventional and holistic therapies, in medicine in general and also in the treatment of cancer. We were not aware of the work by Ryke Geerd Hamer until recently, when his work was discussed at the First International Conference on Holistic Healthcare in Copenhagen, November 1–2, 2004, in connection with the presentation of the first papers from our own holistic cancer healing project[5,6]. This paper is a critical review of the publications of Hamer[1,2,3,4] after reading his material and conducting an Internet and Medline search. Our aim is to examine if the medical principles found by Hamer, what he called his “medical laws”, are substantiated by contemporary holistic medical theory.

## RYKE GEERD HAMER

Ryke Geerd Hamer, MD was born in 1935 and grew up in Frisia, Germany. He started medical and theological studies in Tubingen and at age 20 years, passed the preliminary examination in medicine and completed his theological examinations at 22. At 24 years of age, Hamer passed his medical state examination in Marburg and after his residency 2 years later, he was granted a professional license as a doctor of medicine. Then followed a number of years at the University Clinics of Tubingen and Heidelberg and in 1972 his specialization in internal medicine[1].

Hamer is now 70 years old and his professional life finished. He has not been allowed to practice medicine since 1986. The reason for revoking his license, according to his own biography, was due to his lack of ability to cooperate with the German biomedical physicians[1]. To our knowledge, he is still in prison for practicing without a license even after being sentenced to prison in 1993 and 1997 for unlawful practice of medicine in Germany. Followers of Hamer in Austria and France have been investigated for murder after cancer patients have terminated their prescribed chemotherapy to follow the treatment scheme of Hamer.

Holistic treatment of cancer has become increasingly popular and the Hamer system of holistic cancer treatment has been one of the most popular, but also one of the most controversial. Hamer received his experience from working with more than 10,000 patients[1] and several self-help groups are building on his ideas in Germany, Austria, Italy, and Norway today. Hopelessly sick patients from all over the world study his system to find hope for themselves, while texts, audiotapes, and books by him and about him[1,2,3,4] have been widely distributed and translated into numerous languages. Hamer worked together with his wife, Sigrid, also a physician, in private practice and together they had two girls and two boys. He worked on several projects and created several inventions, like the nontraumatic Hamer-scalpel for plastic surgery (which cuts 20 times more sharply than a razor), a special bone saw for plastic surgery, a massage table that automatically adjusts to the contours of the body, and a device for transcutaneous serum diagnosis. In August 1978, his son, Dirk, was accidentally shot. Dirk’s battle before death lasted for almost 4 months, while his father watched him day and night. It became clear to Hamer, 3 years later, that this sad life event had created an internal shock that he believed resulted in the development of a testicular carcinoma. He worked on understanding his own disease and, during this process, experienced a spontaneous remission. This gave him the understanding that the things he corrected within himself during this process of accelerated personal growth was actually what cured his own cancer. He later named this type of cancer-inducing conflict the "Dirk Hamer Syndrome": a biological conflict or shock that catches one unexpectedly and remains in the body for years leaving it vulnerable to development of disease[1].

In 1981, Hamer thought that these connections applied to cancer and submitted his discovery to the University in Tubingen as a postdoctoral thesis for qualification as a university lecturer. The main objective of the thesis was to provide his results to the University in order to test his hypothesis on patients. In May 1982, the University rejected the work on the interconnections between the psyche and cancer. Over the next few years, Hamer tried repeatedly to open a hospital or clinic as a refuge for his patients so that they could benefit from his knowledge. This was made impossible by court actions against him. In 1986, the District of Koblenz initiated an action to stop Hamer from practicing medicine on the basis that he "failed to deny the iron rule of cancer and failed to convert to the tenets of official medicine." The court also ordered that the University of Tubingen should continue his postdoctoral thesis proceedings. Nothing happened until January 3, 1994, when the judgment to validate Hamer's thesis was executed, but after 13 years, it was unlikely that the University would test his ideas and on the 22nd April, the University announced that "a verification within the framework of the postdoctoral thesis was not planned."

In 2003, branches of the "New Medicine" opened in Oslo and Bergen, where they (through the writings of Hamer) claim that international Jewry knows the cure for cancer and other lethal diseases, but refuses to disclose it in order to exterminate the non-Jews of the world. Hamer has drawn heavily on the racist writings of British New Age conspiracist David Icke, but in this review we will concentrate on his claims for a cure of cancer. The activities of the New Medicine were condemned by the Norwegian Research Council in March 2003.

## A CRITICAL REVIEW OF THE WORK OF HAMER

As it seems that Hamer found something of importance for many patients, we found it of importance to understand how his problems with the academic society and "medical establishment" came about, since it seemed as if he was successful in many cases, highly loved, and appreciated by thousands of his patients. Unfortunately, a Medline search (www.pubmed.gov) showed that we completely lack clinical trials testing his method. From a scientific point of view, Hamer's life and work are interesting and important for the development of scientific holistic medicine. Most of the problems of Hamer's work (seen from the written texts on his work only) seemingly arose from the way Hamer structured his understanding into an idiosyncratic system of holistic healing with five fundamental "medical laws", intending to address the healing of the patient as a whole person, while healing spirit, mind, and body at the same time. Some of these "medical laws" are in agreement with the theories acknowledged by modern holistic medicine such as the theory of coherence by Aaron Antonovsky (1923–1994) that explains that health comes from re-establishing coherence[7,8,9,10,11,12]. This is related to the work and ideas of Abraham Harold Maslow (1908–1970) and Viktor Emil Frankl (1905–1997) and the most progressive resilience literature, as well as our own work: the theory of the purpose of life and the life mission theory that explain that the cause of much suffering and disease results from resignation of the purpose of life[13,14,15,16,17,18,19,20,21]. The simple explanation is that we repress our deep wishes and needs (our self) to adapt to our early environment and our parents; when we do so too radically, we accumulate vulnerability, which becomes an important co-factor in a later development of diseases like cancer. Other medical principles Hamer identified and called "medical laws" unfortunately lack the content and structure that is normally expected from medical science, as they do not acknowledge and incorporate the established knowledge of immunology, toxicology, and other medical fields.

While reading his book, it appears that Hamer was a truly holistic physician: "The most important of everything is that the patient… have obtained new understanding, deep trust in the physician and a real insight in what is going on"[1, p. 45]. In his work, Hamer used the well-known efficiency and healing power of first winning the trust of his patients and then letting the patient do the work of healing himself.

From our perspective, built on many such meaningful statements, his widespread reputation, and popularity among patients, Hamer was a great clinical physician. From our review of his writings, it seems that he was not, however, so great on theory. The lack of an academically acceptable explanation for his work is really very sad. Had Hamer only known more of Hippocrates and the holistic medical histories of Hinduism, Buddhism, and Islam, he would have been much better off referring to these traditions instead of insisting on finding out everything for himself and making his own new system.

On the other hand, we need the wheel of medicine to be reinvented again and again to keep it fresh and useful for the patients of our time and in the actual cultural setting. Hamer did this with great effort and with the intent to benefit his patients. Many of his patients apparently rejected the help they could have gotten from conventional physicians, like chemotherapy and radiation therapy, and turned to Hamer, but that made him open for criticism by other physicians who saw him as responsible for harming these patients. What is stated by Hamer in his book[1] might very well be understood as a warning to the patients towards his biomedical colleagues and thus he might actually be responsible for inspiring some patients to choose not to accept a documented cure and, thus, if not cured by Hamer, dying in spite of the existence of a cure.

We believe that an adult patient must be respected for his autonomy and integrity, but at the same time, a physician must do whatever he can to convince the patient to accept the most rational treatment. When it comes to metastatic cancer, the problem is that there often is little to do, which has a documented clinical significant effect, the NNT (numbers needed to treat) to obtain an effect going up to between 10–20[6,13]. Patient autonomy must therefore (from a medical ethical perspective) be stressed more and the paternalistic position of the physician stressed less. Still, if we as physicians can understand the Hamer system and give advice to the patients about this system, we will be able to form a good dialog with the autonomous and often desperate cancer patient.

In the present work, we have no intention of testing the Hamer system clinically, but only of analyzing it from a theoretical point of view. We want to compare the Hamer system with contemporary theoretical holistic medicine to see what must be acknowledged in his system as true and valuable insight into the mechanism of holistic health and healing and what must be seen as not true (from our present state of knowledge).

In the future, a deeper theoretical understanding of holistic medicine might show that this analysis is unjust to Hamer's system. To make it simple, we have chosen to build this paper on a small book based on interviews with Hamer called "*Cancer — The Riddle That Does Not Exist*"[1] instead of on the very comprehensive and complex presentations[2,3,4] of his work. We believe that an analysis of the five principles or "medical laws" presented as the fundamentals of his holistic system of healing is sufficient for establishing the theoretical value of the Hamer system. In this paper, we use our own wordings of Hamer's last four "medical laws", so as not to confuse the subject with the many idiosyncratic concepts of Hamer.

## Law Number 1: The Hamer "Iron Law of Cancer"

Hamer claims that all cancer forms arise from an emotional and "biological" shock[1, p. 12], causing the patient to retract from the world with a destructive resignation regarding his fundamental wishes. He stresses that this shock must go so deep that it influences the whole biology of the patient's organism and it must go deeper into existence than just the mind. Interestingly, this law is consistent with both Antonovsky's work on coherence[7,8,9,10,11,12] and on our own life mission theory[14,15,16,17,18,19,20,21], which explains development of nongenetic and nontraumatic disease in general and in the same way. Unfortunately, Hamer insists that there are no genetic causes of cancer and that no drug can cause cancer either[1, pp. 49–54]. In his radical insistence on an all-psychological approach, Hamer intimidates a generation of physicians doing research in genetics and the toxicological dangers of smoking. Nevertheless, his "Iron Law of Cancer" (stating the psychosomatic element) seems basically to be in accordance with the works of Antonovsky, Frankl, and our own work in holistic medicine. From a theoretical perspective, we therefore conclude that Hamer's first law of cancer is substantiated.

Interestingly, the process of healing according to Hamer includes a period called the "epileptoform crises" (analogous to an epileptic attack with muscle spasms), where the patient spontaneously regresses to the trauma to integrate this crisis[22]. Only after this incident of healing will the patient improve[1, p. 20]. It is most noteworthy that Hamer observed that the crisis must be sufficiently strong for the patient to heal[1, p. 21]. What Hamer describes here is exactly the same process of healing as described in most work with holistic healing of the patient's whole existence, improving health, quality of life, and ability in general, as explained by the Antonovsky concept of salutogenesis and the holistic process theory of healing[22,23].

The fundamental understanding of the psychosomatic cause of cancer and the ability to win the patients trust and take them into the process of holistic healing of life and existence might very well explain why Hamer's clinical work has been successful for his patients. Claiming that traumas can produce cancer, we can also comprehend (with our present knowledge), but also understand, why he was not well understood and received 20 years ago. As a hypothesis for further research, we would like to see this simple and somewhat provocative statement of Hamer expressed in a little more complex and deeply rooted way to embrace a better understanding of human consciousness[24,25,26,27,28,29,30,31]. Only after decades of theoretical work, and only after we recently have been able to induce similar healing processes with cancer patients in our own research clinic, have we been able to accept and understand the controversial first law of Hamer.

## Law Number 2: Every Disease has a Pathogenetic and Salutogenetic Phase

Unfortunately, Hamer did not know of Aaron Antonovsky[7,8,9,10,11,12] who did his clinical work and constructed his theory of salutogenesis at the same time. Antonovsky simply explained what Hamer observed, making the process of healing the reverse process of the process of pathogenesis (getting sick). Hamer's understanding of pain also seems to be in accordance with the contemporary understanding of pain (physically, emotionally, and existentially) as a necessary part of the process of healing[1, p. 56]. Most importantly, Hamer stressed the importance of solving existential problems in real life, not only in the psyche[1, p. 20]. Understanding the process of healing and being able to take the patient into the process is really what makes a good holistic physician. From the success of Hamer with his patients, it seems he was able to do this.

## Law Number 3: Cancer Development Follows a Simple System of Symbolic Transformation from Psyche to Brain and the Organs of the Body

Many holistic physicians and some of the very popular health prophets of our time, like Louise Hay[32], have claimed the existence of such simple systems that can be used to read the mental and spiritual cause of a physical disease. Unfortunately, we have not yet seen such a system. Quite the contrary, it seems from our research that repressed emotional problems can be moved around in the body and resettle wherever it is most convenient for the organism. The chronic state of whiplash-associated disorder is an example of this[33].

So Law Number 3, for which Hamer gives fylogenetic and ontogenetic arguments, seems to be less accurate from our present state of knowledge. Still, there might be a considerable symbolic element in the disease making the patients able to "listen to the body", but not as schematic as Hamer believed, although we must admit that there could actually be such a symbolic psychosomatic system working in our organism, only with a more complex and not yet discovered set of rules. This is also an important hypothesis for further research.

### Law Number 4: Bacteria and Virus are Controlled by the Body and Help the Body in the Process of Healing

This law seems in complete contradiction with our present knowledge of immunology, so it is not likely to be true. The reason for this understanding seems to be the benefit for the patient of going deeply into the salutogenic crisis, which often is taking so many resources from the patient that (s)he will get an opportunistic infection.

### Law Number 5: All Diseases are Rational and for the Benefit of the Patients

Hamer agues thus from an evolutionary and possibly teleological perspective. We have not found contemporary knowledge to support this law.

## DISCUSSION

Ryke Geerd Hamer wanted his peers to acknowledge his discoveries as hard science. He therefore used the CT scanner to make images of the brain and found that circular patterns (well known as artefacts from the CT scanner) carry vital information on the process of disease and healing. After studying the patterns for years, he claimed that visual pattern, which he then called the “Hamer Herd” or “Hamer Focus” (the German word “herd” means “hearth”, the central place of fire in the house) was always present in the CT scan of a cancer patient’s brain in the pathogenic phase, revealing the path to healing for this patient. The Hamer focus looks like concentric circles around the part of the brain that represented the sick organ in Hamer’s interpretation.

There is a slight possibility that the Hamer focus is actually a great new scientific discovery. It is much more likely to be an artefact that Hamer, for lack of other hard evidence of his theory (which he desperately needed to get his position back in the medical society), gave too much importance. Unfortunately, we do not have the resources necessary to test this part of Hamer’s work. The concentric circles in the Hamer focus, shown on the front page of his book[1], looks like an artefact and very little as a biological phenomena, which in humans are almost never seen as concentric circles. If the center of the phenomena actually is placed in the brain according to the system Hamer described, this must be given further analysis.

In the way we recommend holistic medicine to be practiced[34,35,36,37,38,39,40,41,42,43,44,45,46, 47,48,49,50,51] and understood[14,15,16,17,18,19,20,21,22,23,52,53,54], the use of CT scans and other high-tech tools are not necessary, as the direct communication and emotional contact with the patient gives all the necessary information for the anamnesis and treatment. One of Hamer’s mistakes, in our opinion, might have been to connect what seems to be an important rediscovery of the Hippocratic tradition of holistic treatment used on cancer patients with the CT scan picture, which made it very easy for his peers to ridicule his “spiritistic readings” of the CT images.

The most problematic consequence of this attachment to his third law and the CT scans was his belief that cancers were not able to metastasize[1, p. 47]. He believed that metastases were new cancers developed by the new shocks patients received when they encountered biomedicine. This conviction made him highly unpopular with many biomedically oriented oncologists (cancer physicians) because it made many of his believers avoid conventional physicians. Our own position is the opposite and we believe that modern holistic medicine should acknowledge the well-documented and sad fact that cancers do metastasize, often with the death of the patient as a consequence.

It seems to us that Hamer was too little rooted in the science of biology to make sufficient theories of the highly dynamic picture of cancer he experienced in his clinical practice. On the other hand, biology definitely needs an upgrade to embrace this dynamic[27,28], as already stressed by big thinkers like Nobel laureate (in 1933) Erwin Schrödinger (1887–1961)[55]. Our review of the work of Hamer came to the same conclusion as the Swiss Study Group for Complementary and Alternative Methods in Cancer (SCAC)[56], who found no evidence that most of his assertions were correct, no case of a cure has been published, and an investigation by Der Spiegel through the German authorities identified 50 cancer patients that had been in the care of Hamer and only 7 survived[56]. Still we find that when treated only with psychosocial intervention, a success rate of 15% with this group of mortally ill metastatic cancer patients is remarkable and encouraging for further research.

## CONCLUSION

For decades, Ryke Geerd Hamer has been a controversial figure in holistic medicine with the claim that cancer was a simple thing to heal with holistic medicine. He gave his peers gray hair because of his reference to his five “medical laws”, most of which were not substantiated and some of which were in direct conflict with existing medical theory. It is pretty clear from our analysis that the two most fundamental principles of Hamer’s work (his first and second laws) are well-established principles of holistic medicine today, worded nicely by the Jewish thinker Aaron Antonovsky, but in reality going all the way back to the father of medicine, Hippocrates[57]. Hamer’s understanding of symbols in medicine, virus and bacteria, and the evolutionary process itself differs a great deal from traditional science and we cannot find support for his last three principles or “medical laws” in contemporary holistic medical theory. As Hamer’s understanding of cancer metastasis was built on these failing principles, we suggest that this aspect of Hamer’s thinking (which has been a major reason for controversy) is also not substantiated: Cancer metastasis is not likely to be new cancers induced by the shock the modern biomedical physician gives his patient.

Altogether, it seems that Hamer is in accordance with contemporary holistic medical theory, as the most fundamental principles of his work are built on an understanding very similar to holistic medical thinkers of today and of the past; regarding the most fundamental postulate that cancer patients can be healed by his system of holistic medicine could actually be the case for some of the motivated patients. This must be tested scientifically, however, before being accepted. If proven, we must recommend a rehabilitation of the name and work of Ryke Geerd Hamer. Clinical testing of a cure for cancer based on Hamer’s system must be considered worth the effort; it must be done with physicians trained by Hamer if at all possible.

At the Research Clinic for Holistic Medicine in Copenhagen, we do clinical research to understand how to use Hamer’s first two “laws of cancer”: that we are often damaged by emotionally painful life events making us vulnerable also to the development of cancer and that we can heal by reversing the pathogenetic process into a salutogenic process and regaining biological order[5,6,22,23,33,51,58]. It is of utmost importance that we test and document the effect of such experimental treatments, and we have therefore developed a simple, easy-to-use, and low-cost strategy for documenting holistic healing[59]. We invite the scientific medical community to cooperate in this important new field of evidence-based holistic medicine growing from an emerging scientific understanding of the connections between health, quality of life, and consciousness[31]. We encourage governments and research foundations to give funding in the promising area of holistic cancer treatment a high priority; many patients now want this kind of treatment as it is becoming increasingly popular. The chance of succeeding with the development of a scientific holistic cure for cancer seems fair from a theoretical perspective, and Hamer’s work has pointed out a direction to follow, even if we do not want to use his particular system.

## ACKNOWLEDGMENTS

This study was supported by grants from IMK Almene Fond. The quality of life research was approved by the Copenhagen Scientific Ethical Committee under number (KF)V.100.2123/91.

## BIOSKETCHES

**Søren Ventegodt, MD,** is the director of the Quality of Life Research Center in Copenhagen, Denmark. He is also responsible for a Research Clinic for Holistic Medicine in Copenhagen and is a popular speaker throughout Scandinavia. He has published numerous scientific or popular articles and a number of books on holistic medicine, quality of life, and quality of working life. His most important scientific contributions are the comprehensive SEQOL questionnaire, the very short QoL5 questionnaire, the integrated QOL theory, the holistic process theory, the life mission theory, and the ongoing Danish Quality of Life Research Survey, 1991–94 in cooperation with the University Hospital of Copenhagen and the late professor of pediatrics, Bengt Zachau-Christiansen, MD, PhD. E-mail: ventegodt@livskvalitet.org. Website: www.livskvalitet.org/

**Niels Jørgen Andersen, MSc,** is professor in the Department of Innovation and Economic Organization, Norwegian School of Management. This department conducts research and provides teaching in central topics related to innovation, business development, management of global companies, business history, and economic organization. Research activities within the Department are related to four core subjects within the discipline: business history, cooperative organizations, business development and entrepreneurship, and finally studies of industries with a special focus on the electricity industry. He is also the dynamic chairman of the nonprofit organization Stiftelsen Holistisk Medisin Scandinavia that aims to support the scientific development, research, and documentation of complementary and holistic medicine in Scandinavia. E-mail: niels.j.andersen@bi.no. Website: www.bi.no/users/fgl93013/

**Joav Merrick, MD, DMSc,** is professor of child health and human development affiliated with the Zusman Child Development Center, Division of Pediatrics and Community Health at the Ben Gurion University, Beer-Sheva, Israel; the medical director of the Division for Mental Retardation, Ministry of Social Affairs, Jerusalem; and founder and director of the National Institute of Child Health and Human Development. He has numerous publications in the field of child health and human development, rehabilitation, intellectual disability, disability, health, welfare, abuse, advocacy, quality of life and prevention. Dr. Merrick received the Peter Sabroe Child Award for outstanding work on behalf of Danish Children in 1985 and the International LEGO-Prize ("The Children's Nobel Prize") for an extraordinary contribution towards improvement in child welfare and well being in 1987. E-mail: jmerrick@internet-zahav.net. Website: www.nichd-israel.com
